# Female scent accelerates growth of juvenile male mice

**DOI:** 10.1038/s41598-023-34548-3

**Published:** 2023-05-05

**Authors:** Sarah M. Zala, Brian Church, Wayne K. Potts, Felix Knauer, Dustin J. Penn

**Affiliations:** 1 Konrad Lorenz Institute of Ethology, University of Veterinary Medicine, Vienna, Savoyenstrasse 1a, 1160 Vienna, Austria; 2 Department of Biology, University of Utah, 257 S. 1400 E., Salt Lake City, UT 84112 USA; 3 Research Institute of Wildlife Ecology, University of Veterinary Medicine, Vienna, 1160 Vienna, Austria

**Keywords:** Behavioural ecology, Ecophysiology

## Abstract

Exposing female house mice (*Mus musculus*) to male urinary scent accelerates their sexual development (Vandenbergh effect). Here, we tested whether exposing juvenile male mice to females’ urine similarly influences male growth and size of their sexual organs. We exposed three-week old male house mice to female urine or water (control) for ca. three months. We found that female-exposed males grew significantly faster and gained more body mass than controls, despite all males being reared on a controlled diet, but we detected no differences in males' muscle mass or sexual organs. In contrast, exposing juvenile males to male urine had no effect their growth. We tested whether the males' accelerated growth imposed functional trade-offs on males' immune resistance to an experimental infection. We challenged the same male subjects with an avirulent bacterial pathogen (*Salmonella enterica*), but found no evidence that faster growth impacted their bacterial clearance, body mass or survival during infection compared to controls. Our results provide the first evidence to our knowledge that juvenile male mice accelerate their growth when exposed to the urine of adult females, though we found no evidence that increased growth had negative trade-offs on immune resistance to infectious disease.

## Introduction

Male house mice (*Mus musculus)* deposit urinary scent marks, which contain compounds, often called "pheromones," that attract females (reviewed in^1,2^) and induce changes in female reproductive physiology and behaviour (reviewed in^3,4^). For example, exposure to male urine accelerates female sexual maturation (Vandenbergh effect)^5^, especially the urine of dominant males^6^. Exposure to male urine also promotes oestrous cycling (Whitten effect), sexual receptivity^7,8^ and maternal behaviour^9^. Males increase the excretion of pheromones in their urine once they obtain a territory and become socially dominant^10^, and pheromone production correlates with male reproductive success in seminatural contexts^11^.

The effects of female chemical signals on males are not nearly as well understood, and few female pheromones have been identified (though see^12^). Yet, male mice are attracted to the urine of females^13^ and exposure to female urine elicits male scent-marking^14^ and emission of ultrasonic vocalizations^15–17^, especially if males have previously been exposed to female stimuli (sexually primed)^18^. Exposure to female urine activates a rapid rise in a male luteinizing hormone (LH) and testosterone (hypothalamic-pituitary–gonadal axes or HPG)^19–21^, and stimulates male copulatory behaviour^22,23^ and spermatogenesis^24^. Exposing juvenile males to an adult female mouse accelerates male growth (body mass) and gonadal development (testes mass)^25–28^; however, it is unclear whether these effects are due to female pheromones or to other cues, such as ultrasonic vocalizations (USVs), that potentially have priming effects on the opposite sex^8^.

Maruniak and colleagues (1978) investigated whether exposure to female urine is sufficient to induce male puberty or accelerate growth^27^. They exposed juvenile males (strain CF-1) to adult females between 20 and 36 days of age and confirmed that such exposure promoted male growth and sexual development; whereas exposure to female urine alone had no detectable effects. The authors cautioned against drawing strong conclusions from their negative results, however, and pointed out that "there are many possible methods and patterns of urine exposure that might occur in a natural population but were not tested in these experiments." (p. 254) Thus, studies with wild mice are needed to resolve this question.

Accelerating puberty and growth should enhance male competitive ability, at least up to some optimum^29^ (though see^11^); however, accelerated growth may also have negative tradeoffs^30,31^. For example, faster juvenile growth is correlated with reduced adult lifespans in laboratory mice^32–35^. Male mice exposed to female urine are less able to maintain body mass during a *Salmonella* infection compared to controls^36^. Other studies have found that exposing adult male mice to female-soiled bedding enhanced resistance to influenza (reviewed in^37^); however, it is unclear whether the beneficial effects on resistance to influenza in these studies were due to males' exposure to compounds in female urine or to viral antigens in the bedding. Thus, studies are also needed to test whether increasing the rate of male growth and sexual development have negative tradeoffs on immunity or survival.

Our first aim was to test whether exposure to female urine influences body growth or sexual development of juvenile male mice. We conducted our experiments with wild-derived male house mice (*Mus musculus domesticus*)^36,38^. We exposed newly weaned males (ca. 21 d old) to female urine (or water as control) five times per week for at least 120 d, and monitored their growth. For comparison, we also exposed juvenile males to simulated scent marks of adult males (or water as a control) and monitored their body mass, as co-housing juvenile male mice with an adult male has been found to inhibit their growth and sexual development^26^. Our second aim was to test whether any effects on male growth or sexual development triggered by female urine impairs disease tolerance (resilience) and resistance (pathogen clearance). We challenged male subjects as sexually mature adults with a bacterial infection (*Salmonella enterica* serovar Typhimurium, strain LT2) and monitored their body mass for 14 more days and then assessed pathogen clearance.

## Material and methods

### Animal ethics declaration

The study was carried out in compliance with the ARRIVE guidelines on animal research, all the experiments were conducted at the University of Utah, USA, and were approved by the University of Utah Institutional Animal Care and Use Committee (IACUC, approved protocols 00-02010 and 03-05005) and comply with the laws of the country in which they were performed. All efforts were made to minimize any kind of suffering to the animals.

### Subjects and housing

We used 122 wild-derived male house mice (*Mus musculus domesticus*), maintained in an outbred colony under standard laboratory conditions (F6 generation of wild-caught mice). The wild mice were originally trapped from two 10 km apart locations near Gainesville, Florida (USA)^38^ and systematically bred following lineages to avoid inbreeding. After weaning (ca. age 21–28 d), the mice were singly housed in open type acrylic cages (30 cm × 19 cm × 13 cm) containing cotton nesting material, paper and pine wood chip bedding for environmental enrichment. We provided mice with food (Harlan Teklad Rodent Chow) and tap water ad libitum until 1 month after weaning. Afterwards, males were kept on a controlled food diet (3 to 3.2 g Harlan Teklad Rodent Chow per day – range due to constraints in cutting the pellets, see^36^), until termination of the experiment, as to reduce the possibility that growing mice compensate for increased energy demands by eating more food^39^. The males were kept in a room without females at a constant temperature (22 ± 2 °C) and under a 14:10 h light:dark cycle. The urine donors were sexually mature Swiss Webster mice (32 females and 47 males), which were singly housed, provided with water and food ad libitum*,* and were kept together in one room in a 12:12 h light:dark cycle, and otherwise in the same conditions as above.

### Experimental design and exposure to female urine

We reared 30 pairs of male mice: 16 pairs of brothers and 14 pairs of unrelated males born within the same week. One male in each pair was arbitrarily assigned to the experimental (female urine) and one to the control treatment (water). The mice were born over a period of 3.5 months, thus during our experimental procedure we treated each pair as a blocked unit, providing all the treatments to the experimental and control mice within a pair at the same age. To expose males to female urine, we simulated urinary scent marks by depositing 10 µl of female urine (or water for the controls) onto sterile filter paper (2 cm × 2 cm) and introducing it into each male's cage^14^ five times per week (Monday to Friday), beginning at weaning. We additionally placed clean filter papers (7.5 cm × 7.5 cm) in the males’ cages 2–3 time per week for the duration of the experiment as a new substrate for the males to scent mark. Female urine was collected from 32 adult singly housed female Swiss Webster mice by gently massaging and squeezing their bladder. Urine was stored at –70 °C, and we pooled each individual female’s urine sample over 7 to 20 collection days to control for oestrous state. We chose Swiss Webster due to their larger bladders and easer handling compared to wild-derived mice. We used urine from a different female on subsequent days, as adult males increase their testosterone when exposed to novel females^21^, and to prevent the males from habituating to an individual female's scent. The cages and filter paper of the males exposed to female-urine were visibly more scent-marked than the controls (SMZ and BC personal observation), consistent with previous studies^14^.

We monitored body mass of each subject weekly starting at weaning, and continued until the end of the study. We challenged adult males with *Salmonella* (intraperitoneally at 130 ± 10 d of age (inoculum dosage: 2 × 10^3^ to 2 × 10^4^ cfu/mouse), and assayed pathogen clearance 14 d after inoculation by sacrificing mice (with CO_2_), dissecting their spleens and incubating the bacteria. We also dissected their reproductive organs (testes, epididymis and preputial gland) to obtain their wet mass. For unknown reasons, 10 males died before the *Salmonella* challenge: six males exposed to female urine and four control males exposed to water.

To investigate the effects of female urine-exposure on muscle mass we dissected the males’ left quadriceps and triceps. We obtained dry mass (to the nearest 0.001 g) by washing the muscles in sterile phosphate buffer solution (PBS) and then drying tissues at 59 °C.

### Exposure to male urine

To investigate the effects of male exposure to novel male urine, we conducted the same experiment utilizing male urine as the stimulus and water as control. We used 31 pairs of juvenile male mice: 27 pairs of brothers and four pairs of unrelated males, all pairs were born on the same day. The urine used to expose the experimental mice had been collected before from 47 male adult mice (Swiss Webster) and stored as described above. We pooled the urine of 3–6 males and utilized the urine from different male groups on subsequent days to prevent the experimental males from habituating to the urine. Following the same protocol as before, we *Salmonella* challenged the males at 166 ± 4 days of age (inoculum dosage 2 × 10^4^ cfu/mouse) and determined pathogen loads 14 d after the inoculation. For unknown reasons eight males died before the *Salmonella* challenge (four males exposed to male urine and four males exposed to water).

### Experimental infection and pathogen load quantification

We cultured a strain of *Salmonella enterica* (serovar *typhimurium*, strain LT2), which is avirulent, except in Nramp-1 laboratory mouse strains^40^, in 20 ml of a commercially available brain heart infusion (Becton Dickinson, USA) at 37 °C for 12 h while shaking. We diluted this overnight solution with sterile PBS to the desired concentration and then infected the male mice intraperitoneally (see above). After dissection, we homogenized the whole spleens (in 1 ml of PBS) under sterile conditions. We performed serial dilutions of the spleen homogenates, spread the bacteria on *Salmonella-Shigella* agar plates, and incubated them overnight (37 °C). We determined the pathogen loads by counting the number of colony-forming units per ml of spleen homogenate on the plates (concentration of bacteria per spleen). We used the mean of two replicate plates per mouse.

### Statistical analyses

For analysing the results we used R version 4.2.2^41^, and to estimate changes in body mass over age we applied additive models (library package mgcv)^42^. We modelled treatment (urine exposure or water control) as a fixed factor, age (days) as a treatment-specific spline, and we used the IDs of individuals as random intercepts. At dissection, we tested the effects of stimulus treatments on body growth (i.e., body mass and also tibia length as another index for size) using a multivariate linear model with body mass and tibia as dependent variables and treatment as independent variable. Afterwards, we ran univariate linear models separately for both dependent variables. We investigated treatment effects on reproductive organs (testes, epididymis and preputial gland) and dry muscle mass (left quadriceps and triceps) using linear models with treatment and only body mass as independent variables, since tibia length was not significantly affected by treatment. To test for differences in pathogen load between the treatments we ran linear models with bacterial loads as the dependent variable and treatment and body mass as explanatory variables. We only used body mass as explanatory variable in the female-urine treatment model, as the male-urine treatment did not affect body mass. Results are considered statistically significant at α = 0.05. We provide mean and standard deviations for descriptive statistics, while we provide estimates and standard errors for results from our models.

## Results

### Body mass

**Figure 1:**
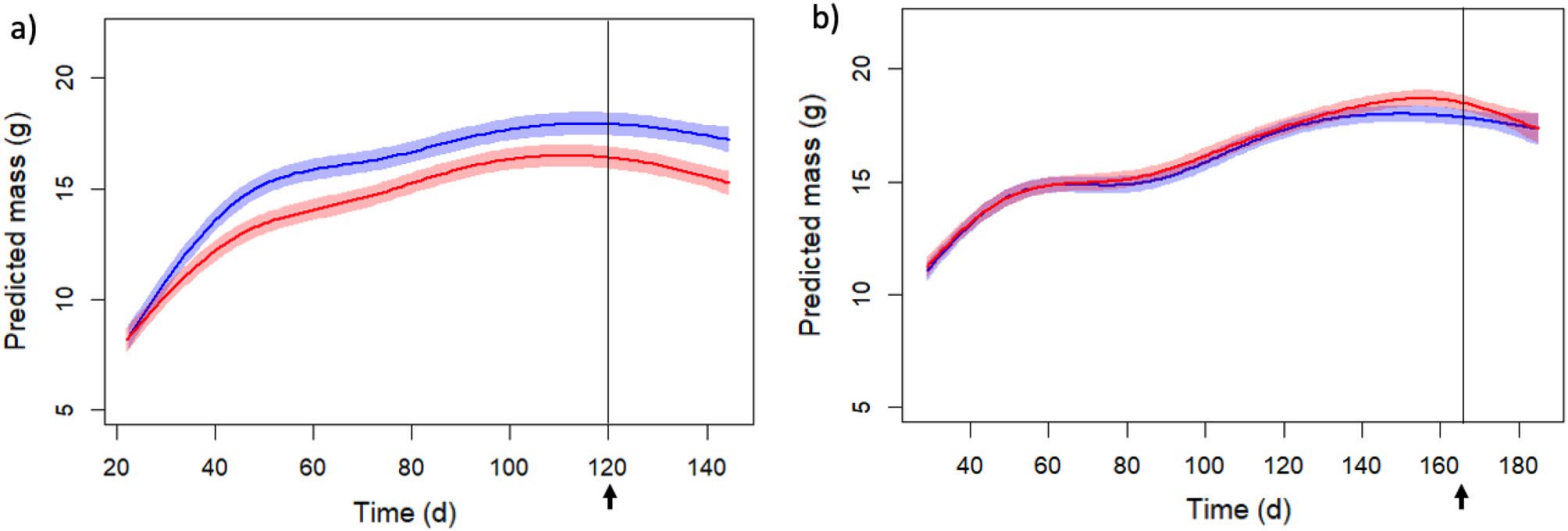
Changes in male body mass over time (age in days) showing the mean mass (continuous line) and 95% confidence intervals (shaded area) for males exposed to (**a**) female urine and (**b**) male urine (blue trend lines) compared to control male mice (red trend lines). Arrows and vertical lines show the mean day of the *Salmonella* challenges.

We found that juvenile males exposed to female urine grew significantly faster and gained more body mass on average compared to sham-control males exposed to water (Fig. 1a, Table 1). The difference in body mass was stable and persisted until the end of the experiment. At dissection, we found that the multivariate model, which included body mass and tibia, was significantly affected by female urine treatment. In the univariate models, only body mass remained significant (16.8 ± 0.4 g female-exposed vs. 15.1 ± 0.5 g controls; *p* = 0.0027). We did not find any effects of treatment on sexual organs (testes, epididymis and preputial gland) or muscle mass after correcting for body mass (n = 42, Appendix 1).

**Table 1:** Effects of female and male urine exposure on male body mass over time.

**Female-urine exposed males**				
	Estimate	SE	t value	*p* value
(Intercept)	15.5663	0.2203	70.645	< 2e−16
Water exposure	− 1.4262	0.3078	− 4.633	4.08e−06

*Approximate significance of smooth terms*				
	edf	Ref. df	F value	*p* value
s(day): urine exposure	5.652	5.95	437.78	< 2e−16
s(day): water exposure	5.584	5.93	338.77	< 2e−16

**Male-urine exposed males**				
	Estimate	SE	t value	*p* value
(Intercept)	3.1608	4.0598	0.779	0.43638
Water exposure	0.2724	0.1025	2.658	0.00794

*Approximate significance of smooth terms*				
	edf	Ref. df	F value	*p* value
s(day): urine exposure	5.6834	5.958	154.465	< 2e−16
s(day): water exposure	5.6818	5.957	175.647	< 2e−16

Female and male urine treatments are compared to water exposure (control). Edf = estimated degrees of freedom; Ref. = reference.

In contrast, exposure to male urine had no effect on juvenile growth (Fig. 1b, Table 1). Individuals in both groups (treatment and control) had a similar body mass, except at a short period towards the end of the experiment and before inoculation (between ca. day 140 to 160), when males exposed to male urine tended to lose body mass compared to the controls. We did not find any effects of treatment on sexual organs (testes, epididymis and preputial gland, n = 40, Appendix 2).

### Salmonella challenge

After infection and dissection, the bacteria load in the spleens of males exposed to female urine (n = 20, log pathogen load 14.4 ± 7 cfu/ml) did not differ significantly from controls (n = 24, log pathogen load 14.4 ± 1 cfu/ml) (*p* = 0.995). Female urine treatment did not affect body mass changes during the *Salmonella* challenge either (Fig. 1a). Six males unexpectedly died during infection (three female-exposed vs. three controls). Similarly, exposure to male urine did not influence males’ pathogen clearance (male urine: n = 19 log pathogen load 11.7 ± 2 cfu/ml versus water controls: n = 21 log pathogen load 11.2 ± 1 cfu/ml) (*p* = 0.711), nor did it affect changes in body mass during the pathogen challenge (Fig. 1b). Twelve males died during infection (eight male-exposed vs. four controls; Binomial-Test, n = 12, *p* = 0.39).

## Discussion

We found that juvenile male mice experimentally exposed to female urine accelerated their growth compared to controls, and female-exposed male mice weighed 1.5 g (9%) more than controls on average. These findings are consistent with previous studies on juvenile male mice reared in the presence of an adult female or exposed to female stimuli through a barrier^26–28^. The effects of female exposure on male growth in these previous studies were mixed or only transitory, whereas our experiment was conducted for a longer period of time and the differences in the males' body mass stabilized until the end of the experiment (> 100 days). We emphasize that these results were not what we originally expected. Instead, we expected that exposing males to female urine would *reduce* male growth, as it increases male scent marking^14^, and scent marking was reported to reduce male growth^43^. In contrast, our results show that males, like females, show pheromone-induced growth acceleration^5^, as previously predicted^27^. They also show that the increased male growth caused by exposure to female urine occurred despite of any increases in male scent marking. Unlike previous studies, however, we found no evidence that exposure to opposite-sex urinary urine enhanced the male sexual organs (testes, epididymis and preputial gland), though we might have missed differences that may have developed earlier before or during puberty, and might have only been temporary. Thus, future studies are needed to examine early effects of female urine on the development of male testes and other organs (epididymis, seminal vesicles and preputial glands)^26–28^.

There were several differences between ours and the previous study by Maruniak and colleagues (1978)^27^: we used different male mice (wild-derived house mice versus CD-1 strain), different feeding protocols (controlled food intake versus ad libitum diet), and different methods for presenting the urine stimulus (10 µl on filter paper inside of males' cages versus 200 µl sprayed on the top). Moreover, we presented urine from a novel individual female, whereas urine in the previous study was presented from a pool of the same females each day, which may have led to olfactory habituation. Such desensitization was another reason that these authors cautioned against drawing conclusions from their negative results. A recently published study on laboratory mice found no evidence that exposure to adult urine affected the growth of either sex^44^, in contrast to a previous study that reported that exposure to the urine of adult virgin females reduced female growth^45^. Interestingly, this recent study found that exposing female mice to the urine of adult females (but not males) increased their longevity (8% in median lifespan), though exposing males to adult urine from either sex had no such effects^44^. Taken together, these studies indicate that exposure to the urine of conspecifics have surprising effects on growth, sexual maturation and even longevity, however, it is unclear how to explain the discrepancies among the studies.

It is not clear how exposure to female urine accelerated male growth in our study, though this effect was not due to having more *access* to food in the present or previous studies, as the mice were kept in cages. Moreover, in the present study differences in food *intake* cannot explain the results, as the males were kept on a controlled diet and usually ate their entire ration each day. Future studies should consider how differences in male growth and puberty might be explained by differences in food intake if the subjects are provided with an unrestricted diet.

Accelerated male puberty and growth induced by exposure to adult females or their urine are likely regulated by endocrine mechanisms, such as increases in the pulsatile secretion of growth hormone (GH) in the pituitary, and insulin-like growth factor (IGF-1) in the liver, which influence puberty and growth^46^. Pheromones in female urine may stimulate male pituitary gonadotropin-releasing hormone (GnRH) transduction, which initiates puberty onset^47,48^. GnRH neurons originate in the olfactory pit, and during development they migrate through the brain to the hypothalamus, where they control the hypothalamic-pituitary–gonadal hormonal axis^49^. GnRH controls the release of luteinizing hormone (LH) from the anterior pituitary, which activates gonadal functions, including testosterone release. Male mice show a dramatic rise in LH release at three weeks of age^50^, which corresponds to the divergence of growth between treatment and control males that we observed. The differences in body mass between treatment males and controls that we observed in our study were significant at the time of puberty (ca. 7 weeks of age), which is approximately when androgen levels peaked in juvenile male mice reared in the presence of adult females^28^. Exposing adult male mice to adult females or female urine elevates male LH levels, which trigger a testosterone surge^19–21^. Maruniak et al. (1978) found that male mice began to secrete LH in response to female urine around 24 to 36 days of age; but repeated exposure to the same female urine resulted in habituation^27^. Thus, studies are needed to investigate the effects of unfamiliar female urine on male GH, GnRH and LH levels, and also pulsatile release, which can now be measured by recently developed methods^51^.

In contrast to exposure to female urine, we found that exposure to adult male urine did not influence the growth of juvenile males, which indicates that growth acceleration in response to female urine is sex-specific. A previous study found that exposing male mice to male urine increased their scent marking, which reportedly reduced their growth rate and body size^43^. An earlier study found that exposing juvenile males to an adult male temporarily inhibited their growth and sexual development^26^; however, these results were likely due to aggressive attacks rather than exposure to adult male urine. Future studies are thus needed to determine which of the many compounds that differ in the urine of male and female mice, both volatile^52^ and non-volatile^53^, explain their different effects on male growth.

We also tested whether exposure to female urine, and any subsequent changes in growth or sexual maturation, impair males' immune resistance to bacterial infection (*Salmonella enterica*); however, we found no evidence for reduced pathogen clearance, body mass, or survival in experimental males compared to control males. Our result show that long-term exposure to female urine does not necessarily impair^36^ nor enhance (reviewed in^37^) male immune resistance to infection. Future studies are needed to test whether more rapid juvenile growth enhances male competitive ability, mating and reproductive success, and if so, whether accelerated growth incurs other trade-offs, such as increased risk of attack from dominant, territorial males^54^.

In summary, our findings show that exposing juvenile male house mice to the urine of sexually mature females triggers increased growth rates and body mass compared to controls. This is the first study to our knowledge to show that exposure to female urine accelerates male growth. The underlying mechanisms are unclear, but endocrine-mediated puberty acceleration is possible. Our findings could prove useful for future studies aiming to manipulate males’ growth or sexual development using more natural methods. Finally, males may accelerate their growth and sexual development when exposed to female stimuli to improve their ability to compete with rivals or attract females. There may be fitness benefits for accelerated growth, and also potential trade-offs, but we found no adverse effects on immune resistance or tolerance to a pathogen challenge. Future studies are now needed to determine whether and how female urine exposure affects male growth and sexual development in a natural or naturalistic social context. Studies are also needed to identify the priming pheromone(s) in female, as well as male urine^55^. Once chemically identified and synthesized, priming pheromones could potentially be applied in agriculture to enhance the growth of livestock species, including domestic swine, sheep, goats and cattle^56,57^, though more natural methods, such as urine, should be sufficient^58^. Such "bio-stimulation" was originally described in ungulates^59^ and first became popular as a method to reduce the age of puberty in domestic swine (boar effect)^57^.

## Supplementary Information

Supplementary Information.

## Data Availability

The data are available through the digital repository of the University of Veterinary Medicine Vienna Phaidra (https://phaidra.vetmeduni.ac.at/detail/o:1236).
