# Supplementary material for: Female scent accelerates growth of juvenile male mice
Source: Sci Rep. 2023 May 5;13:7371. doi: 10.1038/s41598-023-34548-3 (PMC10163255; doi:10.1038/s41598-023-34548-3)
Supplement: Supplementary file 1 — Supplementary Information. [file 41598_2023_34548_MOESM1_ESM.pdf]

Female scent accelerates growth of juvenile male mice

Sarah M. Zala*[1,2], Brian Church[2], Wayne K. Potts[2], Felix Knauer[3] and Dustin J. Penn[1,2]

[1]Konrad Lorenz Institute of Ethology, University of Veterinary Medicine, Vienna, 1160 Vienna, Austria.

[2]Department of Biology, 257 S. 1400 E., University of Utah, Salt Lake City, UT, 84112, USA.

[3]Research Institute of Wildlife Ecology, University of Veterinary Medicine, Vienna, 1160 Vienna, Austria.

* Corresponding author: sarah.zala@vetmeduni.ac.at. Konrad Lorenz Institute of Ethology, University of Veterinary Medicine, Vienna, Savoyenstrasse 1a, 1160 Vienna, Austria.

Appendix 1: Female urine effects on sexual organs(testes, epididymis and preputial gland) and muscle mass(ceps).

```
setwd("C:/…")
dat=read.csv('Data.csv', sep=";")
dat=dat[complete.cases(dat),]
options(na.action = "na.fail")

m=lm(cbind(Testes,Epididy,Prep_gland,ceps)~group+Mass,data=dat)

library(car)
Anova(m)

Type II MANOVA Tests: Pillai test statistic
Df test stat approx F num Df den Df    Pr(>F)
group  1   0.03966   0.3717      4     36    0.8272
Mass   1   0.49932   8.9757      4     36 3.904e-05 ***

summary(m)
```

Response Testes :

```
  Call:
  lm(formula = Testes ~ group + Mass, data = dat)

Residuals:
  Min        1Q    Median        3Q       Max
-0.061249 -0.007303  0.003662  0.012901  0.039125

Coefficients:
             Estimate Std. Error t value Pr(>|t|)
(Intercept)  0.068940   0.043099   1.600   0.1178
group       -0.003465   0.008326  -0.416   0.6796
Mass         0.005439   0.002214   2.457   0.0186 *

Residual standard error: 0.02398 on 39 degrees of freedom
Multiple R-squared:  0.1866,    Adjusted R-squared:  0.1448
F-statistic: 4.472 on 2 and 39 DF,  p-value: 0.01784
```

Response Epididy :

```
  Call:
  lm(formula = Epididy ~ group + Mass, data = dat)

Residuals:
  Min        1Q    Median        3Q       Max
-0.024942 -0.006050 -0.000345  0.005140  0.038696

Coefficients:
  Estimate Std. Error t value Pr(>|t|)
(Intercept)  0.013559   0.019994   0.678   0.5017
group       -0.003631   0.003862  -0.940   0.3529
```

```
Mass         0.002544   0.001027   2.477   0.0177 *

Residual standard error: 0.01112 on 39 degrees of freedom
Multiple R-squared:  0.2268,    Adjusted R-squared:  0.1871
F-statistic: 5.719 on 2 and 39 DF,  p-value: 0.006637
```

Response Prep_gland :

```
  Call:
  lm(formula = Prep_gland ~ group + Mass, data = dat)

Residuals:
  Min        1Q    Median        3Q       Max
-0.021764 -0.012268 -0.003914  0.012525  0.037321

Coefficients:
  Estimate Std. Error t value Pr(>|t|)
(Intercept) -0.043386   0.028687  -1.512  0.13849
group        0.002566   0.005541   0.463  0.64594
Mass         0.004973   0.001474   3.375  0.00168 **

Residual standard error: 0.01596 on 39 degrees of freedom
Multiple R-squared:  0.247,     Adjusted R-squared:  0.2084
F-statistic: 6.396 on 2 and 39 DF,  p-value: 0.003961
```

Response ceps :

```
  Call:
  lm(formula = ceps ~ group + Mass, data = dat)

Residuals:
  Min         1Q     Median         3Q        Max
-0.0104964 -0.0041739 -0.0004844  0.0033078  0.0130136

Coefficients:
  Estimate Std. Error t value Pr(>|t|)
(Intercept) -0.0035368  0.0099772  -0.354    0.725
group       -0.0014289  0.0019273  -0.741    0.463
Mass         0.0022312  0.0005125   4.353 9.39e-05 ***

Residual standard error: 0.00555 on 39 degrees of freedom
Multiple R-squared:  0.4189,    Adjusted R-squared:  0.3891
F-statistic: 14.06 on 2 and 39 DF,  p-value: 2.529e-05
```

Appendix 2: Male urine effects on sexual organs (testes, epididymis and preputial gland).

```
setwd("C:/…")
dat=read.csv('Data.csv')
dat=dat[complete.cases(dat),]
options(na.action = "na.fail")

library(car)

m<-lm(cbind(Testes,Epididy,Prep_gland)~group+Mass,data=dat)
summary(m)

Anova(m)
Type II MANOVA Tests: Pillai test statistic
      Df test stat approx F num Df den Df   Pr(>F)
group  1  0.087539   1.1193      3     35 0.354483
Mass   1  0.311476   5.2778      3     35 0.004144 **
```

Response Testes :

```
  Call:
  lm(formula = Testes ~ group + Mass, data = dat)

Residuals:
  Min        1Q    Median        3Q       Max
-0.031905 -0.010397 -0.003252  0.016754  0.030356

Coefficients:
             Estimate Std. Error t value Pr(>|t|)
(Intercept)  0.054473   0.030258   1.800 0.079973 .
group       -0.007465   0.005852  -1.276 0.210044
Mass         0.006149   0.001559   3.943 0.000345 ***
  ---
  Signif. codes:  0 '***' 0.001 '**' 0.01 '*' 0.05 '.' 0.1 ' ' 1

Residual standard error: 0.01817 on 37 degrees of freedom
Multiple R-squared:  0.343,     Adjusted R-squared:  0.3075
F-statistic: 9.659 on 2 and 37 DF,  p-value: 0.0004215
```

Response Epididy :

```
  Call:
  lm(formula = Epididy ~ group + Mass, data = dat)

Residuals:
  Min        1Q    Median        3Q       Max
-0.047408 -0.022944 -0.009763  0.008007  0.301465

Coefficients:
```

```
               Estimate Std. Error t value Pr(>|t|)
(Intercept)  0.148606   0.092829   1.601    0.118
group       -0.020030   0.017953  -1.116    0.272
Mass        -0.001789   0.004784  -0.374    0.711

Residual standard error: 0.05574 on 37 degrees of freedom
Multiple R-squared:  0.03351,   Adjusted R-squared:  -0.01873
F-statistic: 0.6414 on 2 and 37 DF,  p-value: 0.5323
```

Response Prep_gland :

```
  Call:
  lm(formula = Prep_gland ~ group + Mass, data = dat)

Residuals:
  Min        1Q    Median        3Q       Max
-0.054879 -0.022935 -0.000272  0.021827  0.060288

Coefficients:
             Estimate   Std. Error t value Pr(>|t|)
(Intercept)  0.049353   0.048385   1.020    0.314
group       -0.010465   0.009358  -1.118    0.271
Mass         0.001807   0.002494   0.725    0.473

Residual standard error: 0.02905 on 37 degrees of freedom
Multiple R-squared:  0.05356,   Adjusted R-squared:  0.002403
F-statistic: 1.047 on 2 and 37 DF,  p-value: 0.3612
```
